# The influence of *vgll3* genotypes on sea age at maturity is altered in farmed *mowi* strain Atlantic salmon

**DOI:** 10.1186/s12863-019-0745-9

**Published:** 2019-05-06

**Authors:** Fernando Ayllon, Monica F. Solberg, Kevin A. Glover, Faezeh Mohammadi, Erik Kjærner-Semb, Per Gunnar Fjelldal, Eva Andersson, Tom Hansen, Rolf B. Edvardsen, Anna Wargelius

**Affiliations:** 1 Institute of Marine Research, P.O. Box 1870, Nordnes, NO-5817 Bergen, Norway; 2 Institute of Biology, University of Bergen, Bergen, Norway; 3 Institute of Marine research (IMR), Matre Aquaculture Research Station, 5984 Matredal, Norway

**Keywords:** Atlantic salmon, vgll3, Time at maturity, Age at puberty, Aquaculture, Sex dependent penetrance, Vestigial like protein 3, Genotype phenotype interaction, GxE, Reproduction

## Abstract

**Background:**

In Atlantic salmon in the wild, age at maturity is strongly influenced by the *vgll3* locus. Under farming conditions, light, temperature and feeding regimes are known significantly advance or delay age at maturity. However, the potential influence of the *vgll3* locus on the maturation of salmon reared under farming conditions has been rarely investigated, especially in females.

**Results:**

Here, we reared domesticated salmon (*mowi* strain) with different *vgll3* genotypes under standard farming conditions until they matured at either one, two or more than two sea winters. Interestingly, and in contrast to previous findings in the wild, we were not able to identify a link between *vgll3* and age at maturity in females when reared under farming conditions. For males however, we found that the probability of delaying maturation from one to two sea winters was significantly lower in fish homozygous for the early allele compared to homozygous fish for the late allele, while the probability for heterozygous fish was intermediate. These data also contrast to previous findings in the wild where the early allele has been reported as dominant. However, we found that the probability of males delaying maturation from two to three sea winters was regulated in the same manner as the wild.

**Conclusions:**

Collectively, our data suggest that increased growth rates in *mowi* salmon, caused by high feed intake and artificial light and temperature regimes together with other possible genetic/epigenetic components, may significantly influence the impact that the *vgll3* locus has on age at maturity, especially in females.

In turn, our results show that the *vgll3* locus can only to a large extent be used in selective breeding to control age at maturation in *mowi* males. In summary, we here show that in contrast to the situation in wild salmon, under farming conditions *vgll3* does not seem to influence age at maturity in *mowi* females whereas in *mowi* males, maturing as one or two sea winters it alters the early allele effect from dominant to intermediate.

**Electronic supplementary material:**

The online version of this article (10.1186/s12863-019-0745-9) contains supplementary material, which is available to authorized users.

## Background

Age at maturity represents an important life-history trait that is also key for sustainable Atlantic salmon (*Salmo salar* L.) farming. Early maturation in males causes significant negative impacts in aquaculture due to increased susceptibility to disease, hypo-osmoregulatory problems and significant production losses caused by mortality, impaired growth and downgrading at harvest [1]. Moreover, early maturation may increase the risk of genetic introgression of escaped salmon into wild populations, as early maturing fish that have escaped into the wild are more likely to survive until maturation and attempt to spawn [1]. In addition, breeders may be interested in utilizing genotypes which contribute to shorter generation times. Currently, the problem with early maturation in aquaculture is controlled by artificial light regimes [2]. However, both increasing sea water temperatures associated with climate change, and a growing use of closed farming systems in the marine phase of production, may augment the incidence of early maturation despite the use of photoperiod control [3]. It is therefore important to explore additional options for how maturation can be controlled in salmon farming. For females, early maturation is not a production problem as very few mature as 1SW in farms. However, for breeders it is of great interest to reduce generation time, currently restricted by females, as this decreases the time of introduction of relevant traits to the breeding nucleus.

Recently, a genome region explaining 35–38% of the variation of sea age at maturity in wild Atlantic salmon was discovered. The genomic region found in chr25 harbors three genes, with the *vestigial-like protein* 3 (*vgll3*) gene showing the strongest signature of selection [4, 5]. It is hypothesized that a balancing mechanism between the sexes maintains both the early and late maturation alleles within wild populations, where the allele for late maturation (L) is dominant in females while the early allele (E) is dominant in the males [5]. It should also be noted that other studies suggests a polygenic nature for sea age at maturity, therefore it is plausible that not only environmental conditions and/or *vgll3* affect the trait but also other regions in the genome [6–9]. It has recently been discovered that north American Atlantic salmon populations have a low frequency of the *vgll3* early allele, and instead show a linkage to grilse maturation in a region on chr21(accounting for 6% of the phenotypic variation) instead of chr25 [10]. Furthermore, in European salmon populations selection towards an increase of the *vgll3* early allele has been observed, further illustrating the importance of this genomic region in controlling age of maturation in European populations of wild salmon [11]. Also a recent study has shown a possible function of *vgll3* in Granulosa and Sertoli cells of salmon ovary and testis respectively, as this protein is expressed in these cell types and regulated upon entry into maturity in both male and female salmon [12]. The differential reproductive functions of Sertoli and Granulosa cells in supporting germ cell development may further explain their differential dominance pattern in the wild.

In this study, we investigated how the *vgll3* genotype influences age at maturity in domesticated males and females (commercial *mowi* strain) reared under standard farming conditions. Our results indicate that in females reared under normal farming conditions, *vgll3* genotype is not linked with the age at maturity to the same extent as in famed males or wild females. For males however, and in contrast to previous finding in wild salmon, we found that the probability of maturing at two sea winters instead of at one sea winter was significantly higher in fish homozygous for the late allele compared to homozygous fish for the early allele, while the heterozygous genotype showed an intermediate probability. Under standard aquaculture the probability to delay maturation between two and more than two sea winter males was, like earlier findings in fish found in the wild, significantly lower in individuals homozygous for the early allele and heterozygous than in fish homozygous for the late allele.

## Results

In this study we have followed 2 year-classes of fish of domesticated (commercial *mowi* strain) background (hereon referred to as Y1 and Y2) that were permitted to mature naturally under typical conditions used in commercial aquaculture. We used a binary logistic regression to estimate the predictors for the probability to delay maturation for males and females as a function of the *vgll3* genotype (EE, EL and LL) and the year-class (Y1/Y2), with factor family as random intercept.

**Fig. 1:**
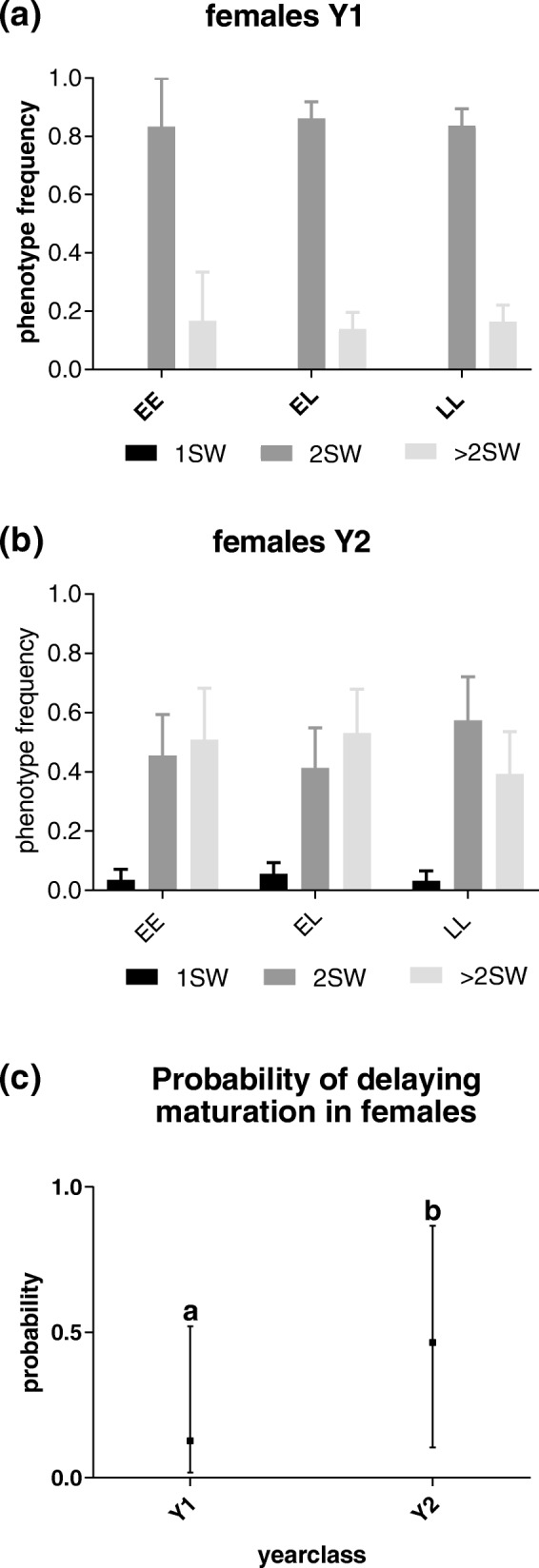
Genotype phenotype interaction in females. Graphs **a**-**b** show phenotype frequencies (1, 2 or > 2SW) per genotype (EE, EL, LL) and family in females (*mowi* strain) reared from two different year-classes (Y1 and Y2, fig **a** and **b** respectively). Graph **c** show the probability of delaying maturation (>2SW, instead of 2SW) when comparing Y1 and Y2 females. Maturation phenotypes are denoted 1SW, 2SW and > 2SW and correspond to fish maturing after 1, 2 or > 2 sea winters (SW). Genotypes are denoted as EE (Early-Early), EL (Early-Late) and LL (Late-Late). Graphs **a-b** shows SEM. Graph **c** shows confidence interval for the probability for delaying maturation, different lower-case letters indicate significant differences (*P* < 0.05)

In females, we analyzed the gene by environment interaction (GxE) of the *vgll3* alleles under standard farming conditions. A clear majority of the females in both year-classes matured as either 2SW (*n* = 211) or > 2SW (*n* = 89), therefore no statistical analysis was performed on the 1SW fish (*n* = 6). Our results indicated that the *vgll3* genotype was not inked to age at maturity in salmon females in either year-class (Fig. 1a-c, Table 1), even though a substantial number of females from each genotype were assayed (Table 2), as well as a considerable number of families. In fact, most females matured as 2SW irrespective of genotype (EE-84% *n* = 50, EL-65% *n* = 127, LL-78% *n* = 128). The, proposed model however, predicted differences between the 2 year-classes (Fig. 1c, Table 1).

**Table 1:** Pseudo R-square estimates. Percentage of the observed variation explained by the fixed effects (marginal) and the combined fixed and random effects (conditional)

Maturation Delaying Probablities	Class	Family	Link	Marginal	Conditional	AIC
Females 2SW to >3SW	glmerMod	binomial	logit	0.1534	0.3589	300.86
Males 1SW to 2SW	glmerMod	binomial	logit	0.1386	0.3143	174.96
Male 2SW to >2SW	glmerMod	binomial	logit	0.4362	0.6044	277.09

**Table 2:** Numbers of fish for each *vgll3* genotype (EE, EL and LL) and phenotype (1SW, 2SW and > 2SW) from year class 1 (Y1) and 2 (Y2)

Phenotype/genotype	Y1			Y2		
	EE	EL	LL	EE	EL	LL
Males						
1SW	10	13	4	7	6	0
2SW	8	54	38	14	12	5
>2SW	1	9	32	15	50	28
Females						
1SW	0	0	0	1	4	1
2SW	13	53	80	14	30	21
>2SW	3	9	13	20	31	13

**Fig. 2:**
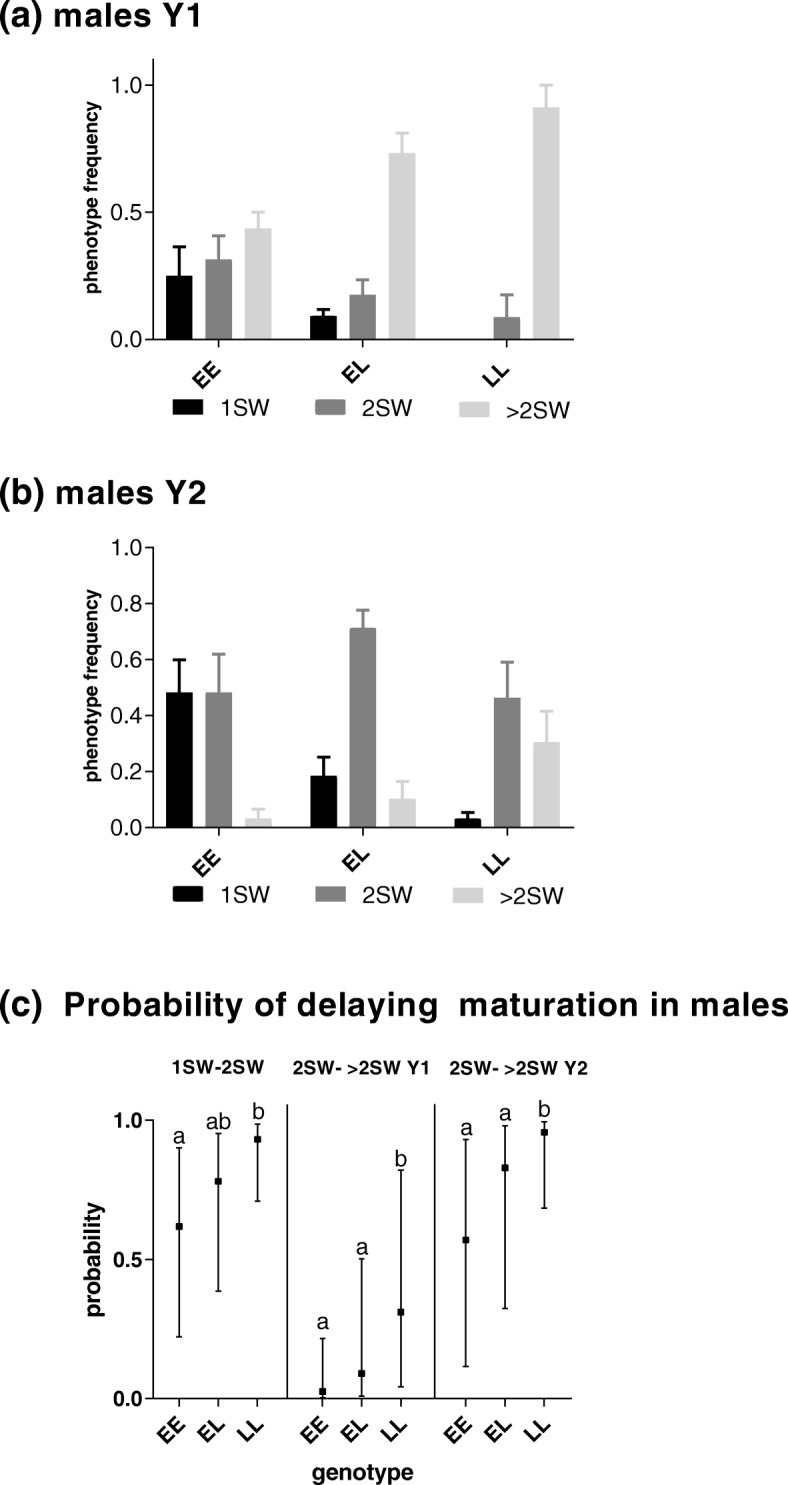
Genotype phenotype interaction in males. Graphs **a** and **b** show maturity frequencies in males (*mowi* strain, 1, 2 or > 2SW) in relation to sea age genotypes (EE, EL and LL) and family in fish from two different year-classes (Y1 and Y2, **a** and **b,** respectively). Graph **c** show the probability of delaying maturation when comparing genotypes (EE, EL and LL) and year-class (Y1 and Y2). Maturation phenotypes are denoted 1SW, 2SW and > 2SW and correspond to fish maturing after 1, 2 or > 2SW sea winters (SW). Genotypes are denoted as EE (Early-Early), EL (Early-Late) and LL (Late-Late). Graphs **a-b** show SEM. Graph **c** shows confidence interval for the probability for delaying maturation, different lower-case letters indicate significant differences (*P* < 0.05)

In males, EE individuals showed a significantly lower probability to delay maturation to 2SW compared to LL individuals. Heterozygous males showed intermediate probability to delay maturation to 2SW, not statistically different from any type of homozygous fish (Fig. 2a-c, Table 1). The probability to delay maturation from 2SW to >2SW was not significantly different between EE and EL individuals in both year classes, while the LL group was significantly different from the other two genotypes.

In both males and females reared in this study, the year-class factor had a significant effect on delaying maturation from 2SW to >2SW (Figs. 1c, 2c, Table 1, Additional file 1). Although the fish were of the same age, Y2 fish were significantly larger than Y1 fish at smoltification, with an average weight and length in Y2 of 223.09 g+/− 61.66 and 26.08 cm+/− 2.64 in comparison to Y1: 90.29 g +/− 19.92 and 19.28 cm +/− 1.45 (Additional file 3).

## Discussion

Understanding the role of *vgll3* in regulating age at maturity in Atlantic salmon reared under farming conditions is important if this gene is to be used to assist selective breeding programs with the aim of controlling maturation of salmon under farming conditions.

The undetectable association between *vgll3* and female age at maturity in females documented herein, contrasts significantly with previous observations in the wild where *vgll3* significantly contributes to age at maturity [5]. It is known that salmon females are strongly affected by environmental cues such as temperature and light, and that this in turn affects time of maturity [2, 13]. In our experiments, juvenile rearing was conducted under continuous light (6 months), the growth promoting effect of continuous light may therefore have affected GxE interaction in fresh water and possible have created an epigenetic mark [14] or affected the endocrine regulation of the brain-pituitary-gonad axis which in turn may have affected age at maturity at sea [1]. The growth spurt under continuous light is also related to a higher feed intake followed by increased growth rate [15–17]. In mice, *vgll3* gene expression has been inversely related to adipogenesis and is thereby indirectly linked to metabolism and energy storage [18]. Interestingly, starvation significantly delays female maturation in Atlantic salmon [19]. Wild salmon also grow slower than domesticated fish both in the wild [20, 21] and under standard farming conditions [22–26], although the difference is much more profound in the latter environment. Our results therefore suggest that a higher growth rate caused by high feed intake and modulated environmental conditions (light and temperature) in *mowi* females, may override the genetic predisposition of the *vgll3* genotype for age at maturity. However, we cannot exclude the possible influence of other genomic-regions or epigenetic effects which may contribute to the observed maturation phenotypes in farmed females and their possible interaction with the environment [5–9, 27–29]. Interestingly some North American strains have a low frequency of the *vgll3* early allele and instead show linkage to grilse maturation on chr21 (6% of the phenotypic variation) instead of chr 25, further showing that other parts of the genome can affect time of puberty in Atlantic salmon [10]. The number of families (*n* = 16) and females per family (*n* = 5–35, average ~ 18) in this study, may have affected the ability of our model to detect weak *vgll3* associations in females. Power analysis for our female model simulating 12 families with up to 15 individuals per family [30], indicated that our model may have limited power to detect genotype effects smaller than the ones detected for males in this study. However, as genotype effects get closer to male values, power to detect effects increase up to 73% (Additional file 1). Based on this finding, our model will most likely detect genotype effects in females similar to the ones reported farmed males herein or for female salmon in the wild [5]. Therefore, *vgll3* genotypes may not be useful to predict nor control the age at maturity in *mowi* females reared under standard farming conditions.

In males, EE individuals showed a significantly lower probability to delay maturation to 2SW compared to LL individuals while heterozygous males showed intermediate probability to delay maturation to 2SWmales. Our result contrasts with earlier findings in wild salmon, where there is a lower probability to delay maturation to 2SW also in heterozygous fish [5]. There may be several explanations for these findings in *mowi* salmon males, both related to domestication genetics, epigenetics and environmental conditions during farming. The most plausible explanation is related to the environment, since it is well known that light regimes efficiently control time of maturity in salmon males under farming conditions [1]. For male salmon, several studies have also shown that prevalence of early maturing fish is lower under feed restricted regimes and in fish with lower lipid stores, clearly linking growth/energy availability to maturity in males [31–35]. A possible genetic difference could be related to the fact that *mowi* salmon have been bred for both late age at maturity and fast growth for nearly 5 decades [24]. In turn, this may have affected the genetic architecture regulating age at maturity. It has been shown that other regions in the genome might contribute to age at maturity in salmon, and possible these regions in the genome are actively controlling age at maturity in the these farmed salmon [5–10, 28] . In our data, the probability to delay maturation from 2SW to >2SW was not significantly different between EE and EL individuals in both year classes, while the LL group was significantly different from the other two genotypes. This observation is consistent with what has previously been observed in wild salmon [5].

In both *mowi* males and females’, Y2 had a significantly higher probability of delaying maturation compared to Y1. It was observed that Y2 fish were significantly larger than Y1 fish at smoltification. Our results indicated that larger smolts tended to mature significantly later (>2SW, instead of 2SW), although it is not possible to conclude whether large smolt size influenced the results due to the large number of other variables which may have caused differences in age at maturity. Nevertheless, it has previously been reported the opposite, that larger smolts are more prone to mature early [36–39]. Interestingly, the year class factor did not have a significant effect on delaying maturation in males from 1SW to 2 SW. The year classes were raised under different environmental conditions, e.g., deviating temperatures, that ultimately could affect freshwater phase growing rates, leading to the observed differences in the influence that smolt size and *vgll3* genotype displayed age at maturity. Further studies using environmental models (temperature, light, and feed restricted or fed to ad libitum) with larger numbers of fish and families are needed to investigate if and how the outcome of the early *vgll3* genotype is affected by growth rate both in fresh and seawater. If the early allele may be more prone to induce earlier maturation in males when juvenile growth rate is low, this should be further explored in production protocols for salmon.

## Conclusions

In conclusion, these data indicate that the *vgll3* genotype does not influence age at maturity in *mowi* females studied herein to the same extent it does for their male counterparts. In *mowi* males, *vgll3* genotype had a significant influence on age at maturity, although different from the wild situation. Therefore, selection for the late *vgll3* allele in this domesticated strain is only likely to reduce the incidence of early maturation in males. Further research on the role of environment, genomic background, and strain specific factors may improve the understanding of these genotypes which may lead to better practice and better marker assisted selection for time of maturity in Atlantic salmon.

## Methods

### Experiments and sampling

The fish used in this study were all reared and sampled at the Institute of Marine Research station at Matre, western Norway. DNA from early (1SW) or late maturing farmed Atlantic salmon individuals (≥2SW) was collected to characterize the *vgll3* genotype. These fish were obtained from two different year classes pedigrees (Y1 and Y2) of Mowi strain. In short, embryos from all families were plucked in equal numbers, 2010 for Y1 and 2011 for Y2. Embryos were subsequently mixed into two “common-garden” replicated tanks for both Y1 and Y2. During start-feeding in common-garden tanks, water temperature was increased to 12–14 degrees, but was dropped down to ambient temperatures approximately three weeks after first feeding had been established. Ambient temperature was not recorded during the remainder of the freshwater stage but varied between ~ 6–14 during the season. During the freshwater stage, fish were reared under continuous light until the late autumn prior to smoltification, upon which they were transferred over to a natural day-length to encourage smoltification as aged 1 smolts in the following spring. This is the typical regime used in commercial farming systems to prevent parr maturation [1]. In spring 2012 and 2013 respectively (Y1 and Y2), length and weight were recorded, and fin clip was sampled for DNA extraction and all fish were PIT tagged for individual identification. Upon smoltification (Aug 16th, 2012 Y1 and May 30th, 2013 Y2), the replicated fish tanks were each placed in individual 5x5x5m sea cages and fish were here reared under ambient seawater and light conditions and checked for maturation once a year until they all reached maturation, this protocol applies to both Y1 and Y2. For details of the production and DNA parental assignment of these families see reference [26, 40]. Before transfer to sea cages, fish were sedated (0.07 gL^− 1^, Finquel, ScanAqua), adipose fin clipped and PIT (passive integrated transponder) tagged. At sampling final sampling, the fish were euthanized (Finquel vet., Scanvacc AS, 0.5 g L^− 1^). Fin clips were preserved on 95% ethanol, from a total of 306 males and 306 females which were used for the final analysis. Full sibling families were represented as follows: Year-class 1 (Y1) consisted of ten families from the *Mowi* strain, with 13, 15, 16, 5, 20, 24, 21, 5, 21 and 31 females and 33, 12, 23, 5, 8, 14, 15, 10, 32 and 17 males per family respectively. Year-class 2 (Y2) consisted of six families from the Mowi strain, in this year-class both males and females were assayed consisting of 10, 36, 12, 31, 13 and 35 males and 6, 34, 15, 27, 25 and 28 females per family respectively. Table 2 summarizes the genetic and phenotypic information of all the individuals used in this experiment (Y1 and Y2).

### DNA extraction, genetic sex determination and genotyping

Total DNA from selected individuals was purified from fin clips using Qiagen DNeasy Blood & Tissue Kit (Qiagen, Hilden, Germany) according to the manufacturer’s recommendations. The genetic sex of all individuals was validated by either a PCR-based methodology (as in [4]) or a DNA probe based RTPCR presence absence assay (Thermo Fisher Scientific, USA) aimed to detect the presence of the male specific *sdY* gene [41, 42].

Two different methodologies were used to determine *vgll3* genotypes of all the individuals at amino acids (aa) 54 and 323. Males reared under standard farming conditions belonging to the Y1 experiment were genotyped on a Sequenom MassARRAY (Agena Bioscience, Germany) as presented in [4]. Genotypes of all fish belonging to the Y2 experiment and females from the Y1 experiment were characterized by and a custom allelic discrimination assay using qPCR (Thermo Fisher Scientific, USA) following the manufacturer’s recommendations. Primers and probes used for sex-determination and genotyping are listed in Additional file 2. Genotype frequencies by sex can be found in Table 2.

### Statistics

Statistics for data arising from the fish experiment were performed in R version 3.4.3., using the *lme4*_1.1–14 and *lsmeans* packages for model selection and validation [43, 44]. All fish used in the model can be found in Additional file 3. Post-hoc tests were performed using the Phia and lsmeans packages (http://CRAN.R-project.org/package=phia) [44]. To investigate effects of the *vgll3* genotypes on the time at maturity binary logistic regression following a Bernoulli distribution was fitted. We tested for the effects of the three *vgll3* genotypes (G), year-class (Y) and the interaction between these as fixed factors. Tank (*t*) and Family (*f*) were included as crossed random intercept factors. The response variable, probability of delaying maturation, was coded as a binary trait with 0 and 1 values and modeled separately for males and females. Thus, to evaluate the probability of delaying maturation between 1 and 2 sea winters, one model was fitted where 1 SW fish were coded as 0 and 2 SW as 1. Another model was fitted where the probability of delaying maturation from 2 to 3 sea winters was coded as 0 for 2SW fish and 1 for >2SW. 1SW maturation was only found in 6 females in total and was therefore excluded from the analysis.

Initial tested model is formulated as follows:

$$ logit\left({p}_{if}\right)=\alpha +{\beta}_1x{G}_{if}+{\beta}_2x{Y}_{if}+{\beta}_3x{G}_{if}x{Y}_{if}+{a}_{\mathrm{t}}+{b}_{\mathrm{t}\mathrm{f}} $$

Where the probability of delaying maturation for individual *i* belonging to family *f* and reared in tank t is a function of the genotype (*G*) and the year class (*Y*) plus the interaction term. Model selection was performed based on the Akaike Information Criterion (AIC) where nonsignificant fixed effects were removed until no further improvement were detected [45]. No significant family variation was detected in the material. To calculate family effects confounding our results, Pseudo R square calculations were performed, to illustrate the percentage of the observed variation by the random effect [46]. Briefly, marginal R^2^ represents the proportion of the total variance explained by the variance in the fitted values for the fixed factors. Conditional R^2^ represents the proportion of variance explained by the variance in the fitted values for the fixed factors and the variance of the random effects from the total variance. For the females, only the year class factor was proven to be significant when delaying maturation from 2SW to >2SW (Table 1, Fig. 1). Genotype was the only factor significant for males when delaying maturation from 1SW to 2SW (Table 1, Fig. 1). We performed power analysis [30] to assess the ability of our female model to detect a range of genotype effect sizes across simulated data sets, consisting of 12 families with a variable number of individuals per family ranging from 1 to 15 (Additional file 1).

## Additional files

Additional file 1: Power analysis. (PDF 108 kb)

Additional file 2: Primer and probes used to interrogate the sex of the individuals (PCR and Presence-Absence) and the *vgll3* genotypes (Sequenom and Allelic Discrimination). (PDF 48 kb)

Additional file 3: A table describing the individual fish number (Pit tag), smolt weight, smolt length, family, sex, sea age at maturity and *vgll3* genotype. (XLSX 36 kb)

## Abbreviations

>2SW: More than two sea winters
1SW: One sea winter
2SW: Two sea winter
aa: Aminoacids
AIC: Akaike Information Criterion
E: Early allele
f: Family
G: Genotypes
GxE: Gene by environment interaction
L: Late allele
PIT: Passive integrated transponder
t: Tank
*vgll3*: *Vestigial-like protein* 3
Y: Yearclass
Y1: Yearclass I
Y2: Yearclass II
