# Supplementary material for: The influence of vgll3 genotypes on sea age at maturity is altered in farmed mowi strain Atlantic salmon
Source: BMC Genet. 2019 May 6;20:44. doi: 10.1186/s12863-019-0745-9 (PMC6501413; doi:10.1186/s12863-019-0745-9)
Supplement: Supplementary file 1 — Power analysis. (PDF 108 kb) [file 12863_2019_745_MOESM1_ESM.pdf]

Additional File 1. Power analysis to confirm the year class effect in females

In this study we did not find any effect of the *vgll3* locus on sea age at maturity, in farmed females from the commercial strain *mowi*. To test the impact of sample size on the ability of the proposed model to detect small size effects (estimates for the fixed factors) we performed simulation-based power analysis. Following Green & MacLeod (2016)[1] we used the three step analysis implemented in SIMR:

1. Simulate a new set of data using the fitted model provided.
2. Refit the model to the simulated data.
3. Apply a test to the simulated fit.

The test will either correctly detect the effect or make a type II error which fails to detect the effect. The power of the test is then calculated as the proportion of successful tests in step three. Since the simulations are independent, the number of successful tests is a binomial random variable, with probability (p) equal to the power of the test, and size (N) equal to the number of simulations, 1000 in our case. Exact confidence intervals for power estimates were used according to Green & MacLeod [1].

We tested the power to detect different effects sizes, namely different estimates or slopes. We used a model with genotype and YC as fixed factors and family and tanks as crossed random effects:

(Intercept): -1.90181163

GenotypeEL: 0.03930722

GenotypeLL: -0.07961167

Ultimate_YCYC2: 1.76479499

We simulated the effect of different estimates for the extreme LL genotype ranging from 1 to 2, similar to the effects detected for males: 2.1 and 2.8 for 1SW to 2SW and 2SW to 3SW maturation delay respectively. The estimates are in the logit scale consequently the impact on the final probabilities will vary as a function of the intercept. For the female data set, 298 individuals from 16 families were used. Of those, 12 families have 15 or more individuals. For each estimated value (1, 1.25, 1.5, 1.75 and 2) we simulated a data set consisting of 12 families with a variable number of individuals per family ranging from 1 to 15.

Our model has a low power to detect effects sizes ≤1.5, ranging from 25% to 45% (**Additional Fig 1**). As estimates get closer to the effect size detected in males for the LL genotype, power increases > 73% for an effect size 2 (**Additional Fig 1**).

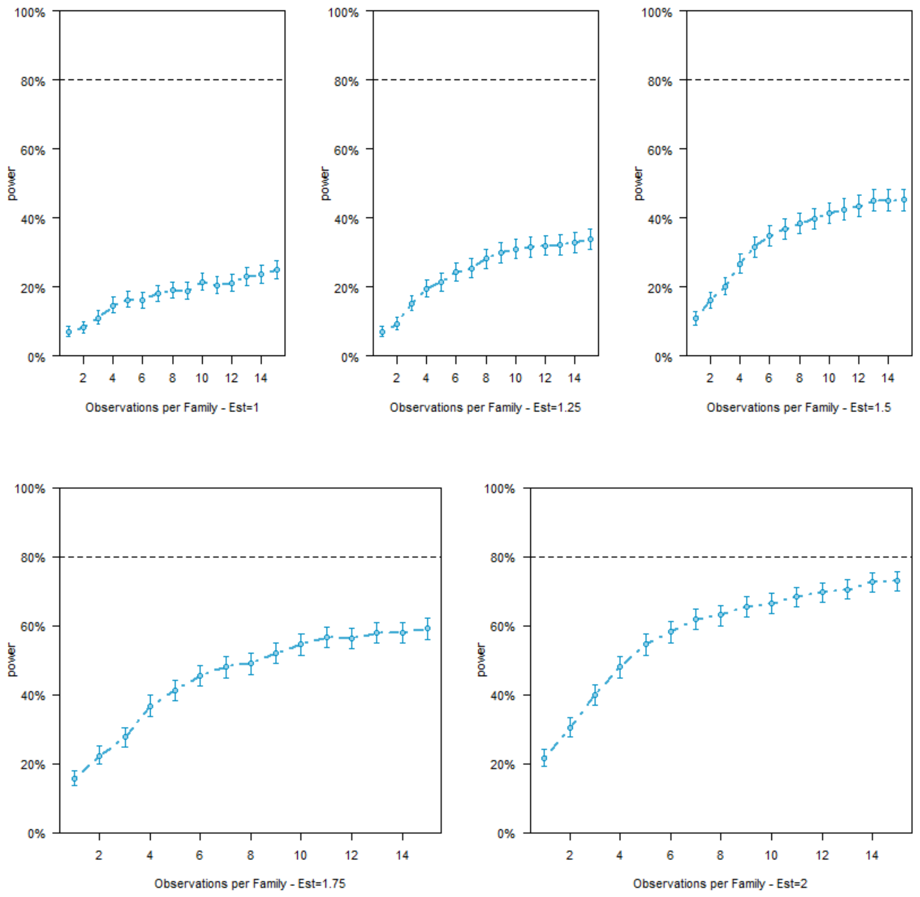

Additional Fig 1: Power analysis with 95% confident intervals to detect estimates (Est) ranging from 1 to 2 for simulated data sets consisting of 12 families and a variable number of individuals per family ranging from 1 to 15 (x axis). Dotted vertical line indicates 80% power.

References

1. Green P, MacLeod CJ: **SIMR: an R package for power analysis of generalized linear mixed models by simulation**. *Methods Ecol Evol* 2016, **7**(4):493-498.
