# Supplementary material for: The influence of vgll3 genotypes on sea age at maturity is altered in farmed mowi strain Atlantic salmon
Source: BMC Genet. 2019 May 6;20:44. doi: 10.1186/s12863-019-0745-9 (PMC6501413; doi:10.1186/s12863-019-0745-9)
Supplement: Supplementary file 2 — Primer and probes used to interrogate the sex of the individuals (PCR and Presence-Absence) and the vgll3 genotypes (Sequenom and Allelic Discrimination). (PDF 48 kb) [file 12863_2019_745_MOESM2_ESM.pdf]

Additional file 2. Primer and probes used to interrogate the sex of the individuals (PCR and Presence-Absence) and the vgll3 genotypes (Sequenom and Allelic Discrimination)

Name	5'-Sequence-3'	Application
sdy_PCR1_F	TGATGGATGGGATCCCCGTCATCTCTCTCCCAAAG	PCR
sdy_PCR1_R	TAGAGCTTAAAACCACTCCACCCTCCATGAGGGA	PCR
sdy_PCR2_F	AGTTGGAACGCTTCAGCAGAGCAGATGG	PCR
sdy_PCR2_F	AGATTGGTGCACTGAGTGATGAGTCTTGTCC	PCR
sdy_RTPCR1_F	CCT ACA AGC CCT TCT CCC TGA T	Presence-Absence
sdy_RTPCR1_R	GGG CTT TGG GAG AGA GAT GAC	Presence-Absence
sdy_RTPCR1_P	ATG GAT GGG ATC CC	Presence-Absence
sdy_RTPCR2_F	CCATGGGCTCAGCAGCTATT	Presence-Absence
sdy_RTPCR2_R	GGAGGACTCAAGCCAGATCCT	Presence-Absence
sdy_RTPCR2_P	AAGCAAGCTCACGACTT	Presence-Absence
ssa25_28656101_SEQ_F	acgttggatgAGCTGGGTGTTTACAGTAGG	Sequenom
ssa25_28656101_SEQ_R	acgttggatgACGCTGCTGTTGCTGTCTC	Sequenom
ssa25_28656101_UEP_SEQ	ccctCCTGGAAACTGCTGCTCC	Sequenom
ssa25_28658151_SEQ_F	acgttggatgAGCCCAGGGATACACAGTGA	Sequenom
ssa25_28658151_SEQ_R	acgttggatgCATGGGTGTGTGTAGAGCAG	Sequenom
ssa25_28658151_UEP_SEQ	GGCTGGCCTGCTCCACCTCTGT	Sequenom
chr25_28656101_AD_F	GAAGCTGGGTGTTTACAGTAGGAT	Allelic discrimination
chr25_28656101_AD_R	TGTCTCCGCCCTGGAAAC	Allelic discrimination
chr25_28656101_AD_V/M	TGCTGCTCC(A/G)TGCTGT	Allelic discrimination
chr25_28658151_AD_F	AGCCCAGGGATACACAGTGA	Allelic discrimination
chr25_28658151_AD_R	GTGGGCCAGGCTGAGG	Allelic discrimination
chr25_28658151_AD_V/M	CCACCTCTGT(G/C)TTCACA	Allelic discrimination
